# Mushroom Penetrating Keratoplasty: A Narrative Review

**DOI:** 10.3390/jcm14072351

**Published:** 2025-03-29

**Authors:** Pietro Bergamaschi, Linda Marie Louise Busin, Angeli Christy Yu, Massimo Busin

**Affiliations:** 1 Department of Translational Medicine, University of Ferrara, 44121 Ferrara, Italyangelichristy.yu@unife.it (A.C.Y.); 2 Department of Ophthalmology, Ospedali Privati Forlì, 47121 Forli, Italy; 3 Istituto Internazionale per la Ricerca e Formazione in Oftalmologia (IRFO), 47122 Forlì, Italy

**Keywords:** cornea, penetrating keratoplasty, mushroom keratoplasty

## Abstract

While full-thickness penetrating keratoplasty (PK) has seen a decline in favor of partial-thickness techniques like endothelial keratoplasty (EK) and anterior lamellar keratoplasty (ALK), PK remains indicated for corneal disease involving the entire corneal thickness. Mushroom keratoplasty (MK) is a noteworthy modification of PK, designed to combine the refractive benefits of a large-diameter anterior lamellar graft with the graft survival advantage of limited replacement of the corneal endothelium. Leveraging the use of a microkeratome, the MK donor graft can be prepared by microkeratome dissection, thereby achieving a central interface compatible with 20/20 Snellen vision. This review explores the various surgical techniques, visual outcomes, graft survival rates, and complication rates associated with MK for various indications.

## 1. Introduction

The history of corneal transplantation dates back to the 1800s, with attempts at using xenograft transplantation as early as 1838. The first penetrating keratoplasty (PK) was performed by Eduard Zirm [1]. Over time, the field of corneal surgery has witnessed considerable advancements. The introduction of the operating microscope enhanced surgical precision, while viscoelastic devices improved intraoperative handling and the protection of the corneal endothelium. Improvements in suture materials as well as the development of corneal trephines, punches, microkeratomes, and even lasers, have further advanced the surgical technique [2].

For much of the 20th century, full-thickness PK was the dominant corneal transplantation technique. The straightforward concept of creating a perfect disc to fit into a perfectly round hole allowed for broad applicability across a range of corneal pathologies, contributing to its widespread adoption [3]. However, PK, as an open-sky surgery, presented several inherent challenges and complications, including significant intraocular complications, poor refractive outcomes, high regular and irregular astigmatism, poor wound healing, delayed visual rehabilitation, immune rejection, and endothelial cell loss. These challenges have generated interest in alternative techniques that offer more targeted surgical treatment [2,3,4].

Specifically, in the last two decades, there has been a dramatic shift in surgical trends toward selective lamellar keratoplasty. Endothelial keratoplasty (EK) has become the gold standard for treating endothelial disease, while deep anterior lamellar keratoplasty (DALK) is preferred for diseases affecting the corneal stroma [5]. These techniques offer better outcomes in terms of faster visual recovery, reduced risk of rejection, and fewer complications. Consequently, PK is no longer currently used for most cases requiring corneal transplantation [6].

However, PK still holds a crucial role in the surgical repertoire. It is particularly indicated in cases of visually significant corneal opacification involving the full thickness of the cornea, where lamellar approaches may not be feasible or successful. In such scenarios, PK remains the definitive procedure to restore visual function, demonstrating its continued relevance in modern ophthalmic surgery [7].

One of the significant modifications in PK is shaped keratoplasty [7,8]. Particularly, mushroom keratoplasty (MK), consisting of a larger-diameter anterior and a smaller-diameter posterior aspect was developed to optimize both the refractive and immunologic outcomes of a full-thickness corneal graft. MK represents an alternative to conventional PK for the treatment of corneal disease involving the stroma, Descemet membrane, and endothelium. This review explores the various surgical techniques, visual outcomes, graft survival rates, and complication rates associated with MK for various indications. 

## 2. Materials and Methods

This narrative review includes only original articles and interventional case series whose main aim was to evaluate the outcomes regarding MK. The review included comparative and non-comparative studies. Meta-analyses and systematic and narrative reviews were excluded.

A literature search was performed using the PubMed library based on the following search terms: (mushroom keratoplasty) OR (mushroom graft) published between January 1950 and the present. There were 581 outcomes in the search, of which 19 articles were selected as relevant after screening based on the aforementioned eligibility criteria by title and abstract.

## 3. Review

### 3.1. History of Mushroom Keratoplasty

**Figure 1:**
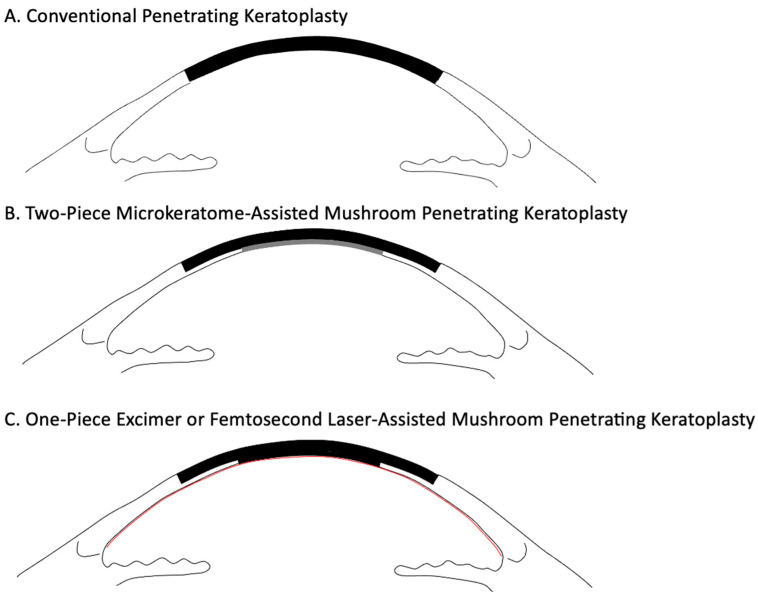
Schematic diagram of penetrating keratoplasty (**A**), two-piece microkeratome-assisted mushroom keratoplasty (**B**), and one-piece excimer or femtosecond laser-assisted mushroom keratoplasty (**C**).

The use of shaped grafts as alternatives to conventional full-thickness PK (Figure 1A) was first proposed in 1921 by Ebeling and Carrel [9]. Early manual techniques faced challenges due to the complexity of the surgical dissection and the limited advantages in comparison with standard PK. Franceschetti, who devised a rudimentary medical cutter, along with early adopters such as Keates, Martinez, Paton, and Roberts, faced difficulties with the technique, which led to its abandonment [10,11,12,13,14,15].

At the turn of the 20th century, the development of the microkeratome facilitated semi-automated dissection of donor corneal tissue. Specifically, Busin introduced a two-piece microkeratome-assisted MK (Figure 1B) wherein following the dissection of the recipient bed, the microkeratome was employed for dissecting the cornea to yield a large 9 mm anterior lamella and a small 6 mm posterior lamella [7,8].

More recently, the use of lasers has been explored for mushroom-shaped grafts. The results of currently used techniques including microkeratome-assisted, excimer laser-assisted, and femtosecond laser-assisted MK (Figure 1C) are discussed in this review.

### 3.2. Concept of Mushroom Keratoplasty

Small full-thickness grafts tend to have longer survival rates than larger grafts in vascularized corneas, due to a lower antigenic load with a lower risk of immune rejection. However, the visual outcomes of smaller grafts are poorer due to resulting high degrees of irregular astigmatism. In contrast, the survival of larger penetrating keratoplasty grafts (7.5 to 8.5 mm in diameter) in corneas with vascularization is often compromised by immunologic rejection and, in post-infectious cases, a recurrence of infection. Owing to its inherent design, MK offers significant advantages over traditional penetrating keratoplasty (PK) [7]. The large-diameter anterior “mushroom hat” combined with a smaller-diameter endothelial replacement “stem” provides improved refractive outcomes with lower risk of immune rejection and subsequent graft failure [8].

Because MK allows the preservation of at least two-thirds of the otherwise healthy recipient endothelium, the recipient bed retains a substantial reservoir of functional endothelial cells, which, when necessary, can migrate onto the posterior surface of the MK graft [8]. Although this concept of minimal endothelial transplantation has not previously been well appreciated, currently there is increasing evidence that even corneal endothelial cells from eyes with endothelial disease, such as Fuchs endothelial corneal dystrophy, are capable of centripetal migration [16,17]. This mechanism of corneal endothelial migration is exploited in more recently introduced interventions such as Descemet stripping without endothelial keratoplasty (DWEK) or Descemet stripping only (DSO), which essentially involves removal of the central Descemet membrane–endothelium complex in early-stage cases of Fuchs endothelial corneal dystrophy [18,19]. The peripheral endothelial cells of sufficient reserve have been found to repopulate the bare central posterior stroma after Descemet membrane stripping and to allow eventual corneal clearance [20].

More recently, the concept of minimal endothelial transplantation has also been adopted in Descemet membrane endothelial keratoplasty (DMEK) through the so-called quarter-DMEK technique. Quarter-DMEK essentially involves transplantation of smaller-sized DMEK grafts for the treatment of Fuchs endothelial corneal dystrophy. In an interventional case series of 12 eyes of 12 patients, Birbal et al. did not observe allograft rejection or graft failure following quarter-DMEK over a 2-year follow-up [21].

While MK is typically performed using the open-sky approach as in a conventional PK, MK minimizes the full-thickness opening to the central 6.0 mm. If there is increased vitreous pressure intraoperatively, the large anterior lamellar graft can be simply fixated with four cardinal sutures, thereby effectively sealing the wound and successively allowing implantation of the smaller (6.0 mm) posterior lamella employing the pull-through technique as in the Descemet-stripping automated endothelial keratoplasty (DSAEK) procedure. This further confers safety of the open-sky procedure [22].

In contrast to the vertical wound configuration in a conventional PK, the step-like wound configuration of the mushroom graft results in an increased surface area of donor-host stromal contact, which theoretically improves wound strength and allows earlier suture removal [8].

Additional procedures can be performed concurrently during the MK procedure, including pupilloplasty, synechioloysis, as well as combined lens procedures including extracapsular cataract extraction and posterior chamber IOL implantation, anterior chamber IOL explantation and scleral-fixated IOL implantation, secondary IOL implantation, and artificial iris IOL implantation [22]. Cataract surgery with monofocal toric intraocular lens implantation after MK can further improve visual outcomes [23].

### 3.3. Two-Piece Microkeratome-Assisted Mushroom Keratoplasty for Various Indications

As initially described by Busin and Arffa [7], two-piece microkeratome-assisted MK can be performed as follows: After performing an initial 9 mm diameter trephination and anterior lamellar dissection, the central 6 mm optical zone is excised to full thickness. The donor cornea is split into anterior and posterior lamellae using a 250 mm microkeratome head and punched to 9 mm and 6 mm, respectively. The posterior donor stem is then placed into the central hole of the recipient bed without sutures, and the anterior lamellar head is on top and sutured with Nylon 10-0 sutures [8].

The two-piece microkeratome-assisted MK can allow proper alignment of the 6.0 mm mushroom stem to the patient’s optical zone [8]. The anterior lamella can be centered on the limbus, while the posterior lamella is centered on the pupil, thus achieving optimal fitting even in eyes with rather eccentric pupils [8].

Similar to laser in situ keratomileusis (LASIK), microkeratome-assisted dissection in MK produces a smooth, regular interface that supports excellent visual outcomes. In terms of technical complexity, MK does not require surgical skills that are different from conventional PK. The additional instrumentation required in MK is limited to the microkeratome. Dissection of the recipient bed may be considered technically challenging, especially in corneas with previous ulcers (i.e., postherpetic scars). However, a perfectly smooth surface is not required, since this step is confined to the peripheral, non-optical region of the cornea. In fact, some “roughness” in the annular zone of contact between the anterior donor lamella and the recipient bed can theoretically enhance healing and possibly contribute to greater wound strength [8].

Furthermore, MK offers several advantages over any anterior LK, including its reproducibility, the possibility of standardization, and the feasibility in eyes with ruptured (i.e., previous hydrops or perforating trauma) or centrally scarred (i.e., previous infections) Descemet membrane.

Table 1 outlines the various surgical indications of MK. 

#### 3.3.1. MK for Vascularized Scars

In cases associated with inflammation and extensive corneal neovascularization, deep anterior lamellar keratoplasty (DALK) is considered be the ideal surgical approach to decrease the risk of recurrence of infection, immunologic rejection, and corneal graft failure. However as corneal scars of infectious origin can affect the Descemet membrane and endothelium, DALK may need to be converted to PK. Our preference is to employ a two-piece microkeratome-assisted mushroom PK in cases of intraoperative complications such as Descemet membrane perforation or cases without satisfactory clearance of the central optical zone. Even in high-risk cases with vascularized corneal scars resulting from various infections, MK confers the refractive benefits of large-diameter grafts with the high survival rate of smaller-diameter full-thickness grafts [24,25,26].

Particularly, in an interventional case series including 31 eyes (herpes simplex virus keratitis *n* = 16; bacterial keratitis, *n* = 10; Acanthamoeba keratitis, *n* = 5) that underwent MK for infectious corneal scars, 83.8% of the eyes achieved a Snellen best corrected visual acuity of 20/40 or better 24. Overall graft survival at 3 years was 90%, which improved to 97% when excluding non-immune corneal graft failure [24].

In a prospective interventional cases series reporting the 10-year outcomes of 52 high-risk cases with at least 2 quadrants of neovascularization that underwent the 2-piece microkeratome-assisted MK with a standard antiviral prophylaxis protocol [25], preoperative best corrected visual acuity (1.73 ± 0.67 logMAR) significantly improved annually during the first 2 years and remained stable up to 10 years. Endothelial cell loss based on central specular microscopy within the donor graft was 41% at 1 year. The average decline of ECL in our series stabilized at approximately 50% of the preoperative value, which is much greater than the minimum ECD levels required for corneal function and transparency. Based on the Kaplan–Meier curve over a 10-year follow-up, the risk of immune rejection, HSV recurrence, and corneal graft failure was 9.7%, 7.8%, and 7.6%, respectively [25]. Since herpetic reactivation can be immunologically triggered by local trauma during surgery, antiviral prophylaxis is considered the standard for prevention of infectious recurrence following MK. Our preference is to initiate high-dose antiviral and steroid prophylaxis with an extended taper. Over a ten-year follow-up, MK for HSV keratitis has been shown to result in significantly improved outcomes with excellent visual outcomes, early ECL stabilization, as well as low rates of herpetic recurrence, immune rejection, and graft failure [25].

In a study comparing the outcomes of DALK and converted MK for herpetic corneal scars [26], DALK was successfully completed in 98 of 120 eyes (81.7%). Of the cases, 18% (22 of 120) required intraoperative conversion to MK. In terms of best spectacle-corrected visual acuity and refractive astigmatism, no significant difference was found between DALK and MK over a five-year follow-up [26]. Expectedly, endothelial cell loss was higher following MK than following DALK. At the 5-year follow-up, the cumulative risks for immunologic rejection (DALK: 3%, MK: 5%, *p* = 0.38), herpetic recurrence (DALK: 6%, MK: 9%, *p* = 0.38), and graft failure (DALK: 4%, MK: 5%, *p* = 0.75) were comparable. The low rates of immunologic rejection, herpetic recurrence, and endothelial cell loss likely contributed to the high overall 5-year survival, which was 96% for DALK and 95% for 2-piece MK [26].

**Figure 2:**
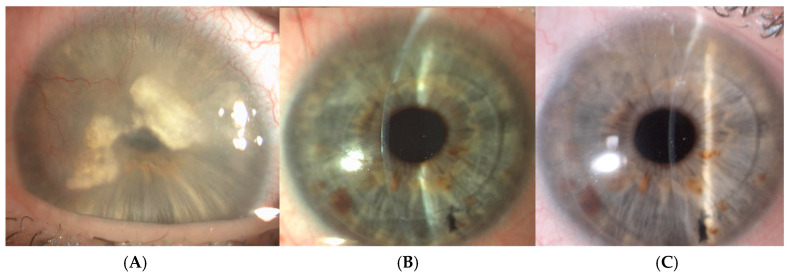
Preoperative clinical picture of a full-thickness herpetic corneal scar with extensive neovascularization. (**A**) Five years postoperatively, the corneal graft is perfectly clear and well adapted, with Snellen visual acuity of 20/20. (**B**) Ten years postoperatively, the graft remains clear, with vision maintained throughout the follow-up period (**C**).

In two-piece MK, the larger 9 mm anterior lamella (the mushroom “hat”) may be anatomically closer to the limbal vessels, which could theoretically increase the risk of immunologic rejection. However, an increased incidence of stromal rejection following MK has not been observed, even in high-risk eyes. MK is an effective surgical option with high survival rates and favorable visual outcomes, particularly in high-risk cases involving vascularized corneal scars following infection (Figure 2A–C) [24,25,26].

#### 3.3.2. MK for Penetrating Trauma

Traumatic scars are typically associated with otherwise healthy corneal endothelium. Of note, such patients are often emmetropic in the fellow eye, meaning the decision to perform any form of corneal transplantation can be difficult to justify without employing a surgical technique that not only can optimize visual restoration but also provide long-term graft survival.

In a study by Yu et al. including 41 cases with traumatic corneal scars that underwent 2-piece microkeratome-assisted MK, Snellen best spectacle-corrected visual acuity was ≥20/100 in 38 eyes (93%) eyes, ≥20/60 in 36 (88%), ≥20/40 in 29 (71%), and ≥20/25 in 13 (32%) eyes just 2 years following MK and complete suture removal [22]. Visual outcomes significantly improved from baseline values (1.41 ± 0.73 logMAR) at one year (0.26 ± 0.13 logMAR) and two years (0.16 ± 0.13 logMAR). Endothelial cell loss was 35.1% at 1 year. Within five years, endothelial cell density values began to plateau, with no significant changes over a ten-year follow-up. The 10-year cumulative risk for immunologic rejection and graft failure was 8.5% and 10%, respectively [22].

From a refractive perspective, using a large-diameter (9 mm) anterior lamella (“mushroom hat”) provides a substantial refractive benefit, especially for patients with unilateral disease who typically may not be amenable to using spectacle correction following surgery. Although posttraumatic cases can often be associated with multiple ocular comorbidities and/or a complex postoperative course, a review of the outcomes of two-piece MK in this cohort provides evidence of sustained long-term visual and survival outcomes over a ten-year follow-up [22].

#### 3.3.3. MK for Failed Attempts at Deep Anterior Lamellar Keratoplasty

To confidently perform large-diameter keratoplasty, it is crucial to have a technique that, in cases requiring conversion to PK, preserves the refractive advantages of the initial wide-diameter trephination while reducing the risk of immune rejection and graft failure.

In our center, we perform two-piece microkeratome MK for DALK attempts requiring conversion, such as DM macroperforation or the presence of a full-thickness opacity within the optical zone. This approach allows the safe attempt of 9 mm DALK. Even when conversion to full-thickness grafting is necessary, MK allows excellent outcomes over a long-term follow-up [8].

In an interventional case series by Myerscough et al. including 416 consecutive attempts of DALK for keratoconus, conversion to 2-piece MK was required in 16% of cases [28]. No difference in best corrected visual acuity was observed between the DALK and MK groups over a five-year follow-up [28]. Expectedly, endothelial cell loss was significantly higher in the MK group than in the DALK group. The Kaplan–Meier survival curve demonstrated significantly higher graft survival in the DALK group versus the MK group (*p* < 0.001). Notably, however, the cumulative probability of survival at 2 years remained stable in both DALK and MK over the 5-year follow-up [28]. The excellent clinical outcomes associated with a two-piece MK in cases converted from intended DALK support the use of large-diameter DALK (9 mm) for keratoconus.

Two-piece MK can also be performed in cases of unsuccessful stromal peeling for DALK in post-PK eyes. In these cases, the two-piece graft can consist of a large 9 mm diameter anterior lamella and a smaller posterior lamella of the same diameter as the old PK graft (6.5 to 8 mm) [29,32].

#### 3.3.4. Other Applications of MK

Two-piece MK can also be performed in children [33]. Considering the higher risk of ocular trauma in the pediatric population, the step-wound configuration of the mushroom graft confers greater wound strength. This mechanical advantage of MK is particularly important in preventing traumatic wound dehiscence over the patient’s lifetime [33,34].

Table 2 summarizes the outcomes of various studies on two-piece microkeratome-assisted mushroom keratoplasty.

### 3.4. Application of Femtosecond Laser for Mushroom Keratoplasty 

The use of lasers in MK offers potential advantages due to the precision and versatility in creating customized graft shapes and depths [27,30,31,35,36,37,38,39].

Table 3 summarizes the outcomes of various studies on excimer laser-assisted and femtosecond laser-assisted mushroom keratoplasty.

A series of 15 eyes who underwent excimer laser mushroom-shaped penetrating keratoplasty has been reported over an 11-month follow-up. Endothelial cell loss was 17% at 1 year [27]. While the study showed relative safety, the use of the excimer laser for PK has not been widely used due to the potential impact of the excimer laser on the corneal endothelium related to mechanical trauma from shock waves, oxidation, as well as the thermal effects from ultraviolet light.

The femtosecond laser has also been applied to the MK technique [30,31,35,36,37,38,39]. Ex vivo studies showed that MK-shaped wounds are more resistant to leakage and rupture compared with conventional PK wounds [35]. Small case series employing femtosecond laser-assisted MK for keratoconus (*n* = 6) [31], infectious keratitis (*n* = 7) [36], pediatric eyes (*n* = 8) [30], and repeat keratoplasty (*n* = 4) showed promising results. In a comparative study between 8.5 mm femtosecond laser-assisted MK and 7.5 mm PK, visual outcomes were comparable between the groups, with expectedly lower levels of astigmatism in the MK group [38].

Another study comparing conventional PK (8.0 ± 0.4 mm diameter, *n* = 1254), femtosecond laser-assisted MK (8.3 ± 0.3 mm diameter, *n* = 34), and femtosecond laser-assisted top hat PK (8.0 ± 0.3 mm diameter, *n* = 102) found no differences in visual acuity among the groups. In this study, the MK group demonstrated better optical outcomes, likely due to the larger mean diameter. However, in terms of the percentage of eyes with endothelial cell count greater than 1000 cells/mm^2^, the MK group was significantly lower than the conventional PK group [39]. Thus, the femtosecond laser pulse-induced microphoto disruption cannot be discounted as a contributing factor to a significantly higher percentage of endothelial cell loss.

Moreover, several limitations hinder the effectiveness of the femtosecond laser, particularly when dealing with scarred and irregular corneas. One of the primary challenges is the femtosecond laser’s inability to penetrate sufficiently through nontransparent tissues. This limitation becomes critical in cases involving vascularized corneas or when bleeding is present, as the laser fails to work under these conditions. Moreover, as the femtosecond laser requires applanation of the recipient cornea, the technique can lead to uneven dissection planes if the corneal thickness is not regular, as in cases associated with corneal ulceration or scarring.

MK performed with a microkeratome, excimer, or femtosecond laser incurs additional costs compared with conventional PK due to the need for specialized equipment, with the cost of either the excimer or femtosecond laser being significantly higher due to the added expense of disposables and laser equipment. In our center, we prefer the two-piece microkeratome-assisted MK technique since we have the microkeratome in suite, such that our costs remain similar to those incurred when preparing microkeratome-assisted grafts for DSAEK and DALK. Alternatively, tissue preparation can be outsourced to an eye bank, where the graft can be pre-cut using a microkeratome and delivered as anterior and posterior lamellae similar to the pre-cut DSAEK. 

## 4. Conclusions

Mushroom-shaped grafts are designed to enhance both refractive and immunologic outcomes. By combining the refractive benefits of a large-diameter anterior lamellar graft with the immune advantages of limited endothelial replacement, MK reduces the risk of immune rejection while maintaining optimal visual performance. The use of a microkeratome enables precise dissection of the donor tissue, ensuring a well-aligned central interface conducive to achieving 20/20 vision. While the use of a femtosecond laser in MK has been shown to yield comparable outcomes, this approach comes with the trade-off of increased surgical costs and extended operating time, thereby limiting the compelling need to use the femtosecond laser.

MK is particularly indicated for corneal diseases involving full-thickness damage, such as corneal penetrating trauma, hydrops, and dense scarring. Although vascularized corneal scars are traditionally considered high-risk for graft failure, MK’s approach of limited endothelial transplantation has substantially improved outcomes over long-term follow-up. This includes excellent visual outcomes, early stabilization of endothelial cell loss (ECL), and reduced rates of immune rejection and corneal graft failure, making MK a valuable technique for managing complex corneal conditions.

## Figures and Tables

**Table 1:** Surgical indications of MK.

Surgical Indications of MK	Comments
Vascularized scars [24,25,26,27]	MK is a viable alternative in high-risk cases with vascularized corneal scars resulting from infections like herpes simplex virus (HSV), bacterial keratitis, and Acanthamoeba. It combines the visual and refractive advantages of large PK grafts with the high survival rates of smaller PK grafts. Antiviral prophylaxis is essential in herpetic cases to reduce recurrence and rejection.
2. Penetrating trauma [22]	MK is effective in traumatic corneal scars with preserved endothelium, offering excellent long-term visual outcomes. The large anterior graft diameter optimizes refractive results, particularly in unilateral injuries where contact lens use is not feasible. Long-term graft survival is favorable over long-term follow-up.
3. Failed attempt at deep anterior lamellar keratoplasty [28]	When DALK is attempted for corneal stromal disease but requires conversion due to Descemet membrane perforation or residual opacity, MK preserves the large-diameter trephination benefits while minimizing immune rejection risks. It provides stable outcomes even in cases requiring secondary intervention.
4. Pediatric corneal opacities [29,30]	In pediatric cases, MK provides better wound strength due to its step-wound configuration, reducing the risk of traumatic wound dehiscence. This is particularly important in children, who are at higher risk of ocular trauma over their lifetime.
5. Hydrops secondary to keratoconus [8,31]	Corneal hydrops involves a break in Descemet’s membrane with full-thickness scarring. DALK attempts often require conversion in these cases. Since the peripheral endothelium is usually healthy, MK is a good option to restore corneal clarity while optimizing long-term graft survival.

**Table 2:** Differential outcomes of studies on two-piece microkeratome-assisted mushroom keratoplasty.

Author	Busin et al. [8]	Yu et al. [4]	Myerscough et al. [28]	Busin et al. [33]	Scorcia et al. [24]	Yu et al. [25]
Year of publication	2015	2022	2020	2011	2012	2020
Total MKs in the study	172	41	68	6	31	52
Duration of follow-up	5 years	10 years	5 years	3 years	3 years	10 years
Indications for MK	Various	Posttraumatic scars	KC	Various in pediatric patients	Post-infective scars	Post-HSV scars
BCVA at the 2-year follow-up (logMAR)	0.11 ± 0.17	0.157 ± 0.148	0.10 ± 0.10	-	-	0.17 ± 0.18
Mean astigmatism (D)	3.36 ± 1.09	3.27 ± 1.50	3.27 ± 1.50	2.60	3.10	2.4 ± 1.8
Mean endothelial cell loss at year 2 (%)	41.7 ± 16.9	39.9 ± 15.9	46.24	24	40.7	40.9 ± 24.1
Survival rate at the end of the follow-up (%)	95.3	90.0	94.12	100	96.7	92.4

**Table 3:** Differential outcomes of studies on excimer laser-assisted and femtosecond laser-assisted microkeratome-assisted mushroom keratoplasty.

Author	Del Valle et al. [27]	Fung et al. [31]	Hosny et al. [36]	Elkamshoushy et al. [30]	Levinger et al. [38]	Daniel et al. [39]
Year of publication	2014	2015	2020	2019	2014	2016
Total MKs in the study	15	4	7	8	26	141
Duration of follow-up	11.9 ± 2.7 months	33 months	6 months	12 months	12 months	2.8 ± 1.5 years
Indications for MK	Post-infective scars (*n* = 11), severe KC with Descemet opacity (*n* = 4)	Previous hydrops	Infectious keratitis	Various in pediatric patients	KC	KC
BCVA	0.69 ± 0.24 (Snellen decimal)	0.00 ± 0.08 (logMAR)	0.17 ± 0.13 (logMAR)	0.10	0.31 ± 0.55 (logMAR)	0.20 ± 0.20 (logMAR)
Mean astigmatism (D)	1.8 ± 1.1	3.38 ± 1.59	-	2.60	−2.84 ± 1.08	−5.9 ± 3.2
Mean endothelial cell loss (%)	17% at 1 year	-	-	13.3	32.1	-
Survival rate at the end of the follow-up (%)	100	100	100	100	100	-

## Abbreviations

The following abbreviations are used in this manuscript:

DALK	Deep anterior lamellar keratoplasty
DMEK	Descemet membrane endothelial keratoplasty
DSAEK	Descemet-stripping automated endothelial keratoplasty
MK	Mushroom keratoplasty
PK	Penetrating keratoplasty
